# The Role of Mental Health, Recent Trauma, and Suicidal Behavior in Officer-Involved Shootings: A Public Health Perspective

**DOI:** 10.3390/ijerph22060945

**Published:** 2025-06-17

**Authors:** Liam O’Neill

**Affiliations:** Department of Rehabilitation and Health Services, College of Health and Public Service, University of North Texas, Denton, TX 76203, USA; liam.o’neill@unt.edu

**Keywords:** officer-involved shooting, mental illness, suicide by cop, methamphetamine use disorder, homelessness

## Abstract

This study uses a public health approach to identify the comorbid risk factors and protective factors that influence the likelihood of an officer-involved shooting (OIS). Methods: We analyzed 7.5 years of hospital inpatient data obtained from the state of Texas. The OIS subjects (*n* = 177) were civilians who were shot during a legal intervention involving law enforcement. The control group (*n* = 33,539) included persons who were hospitalized due to injuries from a car accident. Logistic regression models were used to identify the predictors of an OIS incident. The data included information on chronic diseases, vulnerable population status, health insurance, mental health diagnoses, substance use disorders, and recent trauma. Results: About one-fourth (24.3%) of OIS subjects had a diagnosed mental illness, compared to 8.4% of control subjects (*p* < 0.001). Factors that greatly increased the risk for an OIS included the following: schizophrenia (AOR = 2.7; CI: 1.6, 4.6), methamphetamine use disorder (AOR = 3.5; CI: 2.2, 5.5), and recent family bereavement (AOR = 8.5; CI: 1.8, 39.6). Six subjects (3.4%) were persons experiencing homelessness (PEH). Protective factors that lowered the risk for an OIS included commercial health insurance (AOR = 0.27; CI: 0.17, 0.45) and Medicaid insurance (AOR = 0.61; CI: 0.11, 0.93). Conclusions: These findings underscore the preventable nature of many OIS incidents, especially those that involve untreated mental illness, homelessness, substance use disorders, and recent trauma. Addressing the root causes of these incidents will likely require interdisciplinary collaboration among law enforcement, public health agencies, and social services.

## 1. Introduction

Firearm injuries and gun violence are significant threats to public health. Yet policy makers have been constrained by the lack of evidence-based research in this area. Law enforcement shootings have come under intense public scrutiny in recent years, even though they account for less than 1% of firearm deaths and 1.6% of nonfatal injuries [1]. Beyond the immediate loss of life, these events have adverse effects for mental health and may contribute to the erosion of public trust in our institutions.

The aim of this study is to identify both the comorbid risk factors and the protective factors that influence the likelihood for an officer-involved shooting (OIS). We will use hospital administrative data to identify the most likely predictors of OIS for a range of covariates. These include vulnerable population status (e.g., being homeless), chronic disease (e.g., hepatitis), recent trauma, mental illness (e.g., schizophrenia), substance use disorders, and suicidal behavior.

Mental illness has been associated with an increased risk for an OIS incident [2,3]. This finding has been observed consistently across numerous settings and countries. DeVylder et al. (2022) have suggested that police violence may precede and contribute to sub-clinical psychotic symptoms [4]. Therefore, mental illness may be both a cause and an effect, as it relates to police violence. The authors also stressed the need for the systematic tracking of OIS incidents, as well as mandatory reporting across police precincts.

A consistent theme of the OIS literature is how progress in the field has been hampered by the lack of quality data [5,6]. Traditional data sources on OIS incidents have noted limitations, such as missing data due to a lack of reporting requirements. Thus, there is an urgent need for comprehensive, standardized databases that are suitable for OIS research and the development of robust, statistical models [5,6,7,8].

Three types of databases have been used in OIS studies and each of these has strengths and limitations. These include law enforcement, media reports, and health care databases. Law enforcement databases provide detailed information on the circumstances of the OIS incident, such as whether the suspect was armed, type of weapons used, and the number of gunshot wounds. Police reports often rely on interviews with witnesses and family members. Most studies that rely on these data have treated mental illness as a single, generic category, which has limited insights into specific psychiatric conditions. Likewise, drugs and alcohol are often grouped into a single category, such as “subject was under the influence of drugs and/or alcohol” [9].

A systematic review of the OIS literature analyzed 86 articles from 15 countries that were published from 2011 to 2020 [10]. The evidence indicated that persons with mental illness or suicidal ideation were at an increased risk for OIS. However, there was no mention of specific psychiatric diagnoses, such as schizophrenia or bipolar disorder. In addition, there were no reported findings on the role of drugs or alcohol as contributing factors. Yet other studies have found substance use to be a significant risk factor for OIS incidents [3]. Hence, there is a need for additional studies to address these gaps.

Beginning in 2015, multiple sources have collected data on fatal OIS encounters. However, most OIS incidents do not result in fatalities, and nonfatal OISs are often overlooked by the media and policy makers [8]. In addition, uniform standards for reporting nonfatal OIS incidents are lacking. According to a recent review, two-thirds of studies did not report information on nonfatal OIS incidents [10]. As a percentage of all police encounters, an OIS is a rare event. Therefore, it is important to have complete data on all OIS incidents, regardless of whether they resulted in fatalities [8,11,12].

This study addresses these limitations by using hospital administrative data, which offer several key advantages [12].

These data contain up to twenty-four diagnosis codes that were abstracted from patient medical records. These contain information on chronic disease, psychiatric diagnoses, substance use disorders, and vulnerable population status. These diagnosis codes are based on the International Classification of Diseases, Tenth Revision, Clinical Modification (ICD10-CM) and have a high degree of specificity and granularity. Hospital databases also capture both fatal and nonfatal injuries due to legal interventions. This study aims to demonstrate the value of health care databases as a complement to more traditional sources of information on OIS incidents.

Hospital data may also contain information on patients’ housing status, evidence of recent trauma, and other social determinants. According to DeVylder et al., there is a need for further research from a public health perspective on the impact of police violence on marginalized populations, such as the homeless [4]. They also called for additional studies on the mental and physical effects of bereavement and its association with police violence.

A previous study of 812 fatal OISs found that 18 percent of cases were classified as “suicide-by-cop” (SBC) incidents [3]. SBC is defined as a method of suicide wherein a subject engages in threatening behavior in an attempt to be killed by law enforcement [13]. Building on this insight, we include both suicidal behavior and a history of recent trauma as explanatory variables.

In summary, most prior studies on OIS have either excluded mental health and substance use variables or treated them as broad, generic categories [10]. Hence there is a gap in the literature concerning the impact of specific psychiatric diagnoses on the risk of an OIS incident. By integrating detailed clinical data into the study of OIS risk factors, this study seeks to address this gap. In the sections that follow, we describe the data source, define key variables, and outline the statistical methods used to assess associations between clinical and social factors and the likelihood of an OIS.

## 2. Materials and Methods

This study used 7.5 years of hospital inpatient data from the Texas Department of State Health Services. The database contains detailed patient information, such as age, sex, race, and one principal and up to twenty-four secondary diagnosis codes. It also contains up to ten external cause codes (E-codes). These codes provide greater detail about injuries, such as the mechanism of injury (e.g., sharp instrument, handgun), the intent behind the incident (e.g., intentional self-harm, legal intervention), and where the injury occurred (e.g., home, highway). This is a comprehensive database that captures 93% to 97% of all hospital discharges statewide. As the data have been de-identified, it is impossible to identify individuals or link their data to any other information source.

From a data perspective, OIS incidents are vanishingly rare, with a rate of approximately 10 per million hospital inpatients. These patients were identified from ICD10-CM codes, using the E-code prefix “Y35,” which classify injuries due to legal interventions (All of the ICD-10-CM codes used in this analysis can be found in the Supplementary Material). There were 187 inpatients who were treated at Texas hospitals for injuries due to an OIS incident between 1 January 2016 and 30 June 2023. Ten patients were prisoners who were incarcerated at the time of the OIS, and these were excluded from further analysis because this study was focused on civilians. The remaining OIS subjects (*n* = 177) were identified as suspects during a legal intervention involving law enforcement (or others with the legal authority to use lethal force). The database also contains information on 160 law enforcement officers who were injured by firearms during interactions with civilians.

The control group (*n* = 33,539) included persons (age 12 and older) who were hospitalized due to injuries from a car accident during the study period. In order to ensure a broad cross-section of the population, the group included drivers, passengers, pedestrians, and cyclists. Like OIS subjects, persons who are injured in car accidents often experience acute, sudden trauma rather than gradual health decline. This similarity reduces bias introduced by chronic illness and advanced age. Because both types of trauma are unplanned and involve emergency medical services, they present similar profiles in terms of health care access and urgency of care. This methodological approach is also consistent with a recent study on the risk factors for suicide by firearm [14]. The researchers used persons who died in motor vehicle accidents as the control group, since in both cases, the deaths were sudden and unexpected.

Vulnerable populations were identified based on the following categories: people experiencing homelessness (PEH), persons with a disability, persons with wheelchair dependence, persons with post-traumatic stress disorder (PTSD), and bereaved persons, who have experienced the recent death or disappearance of a family member. Unless otherwise noted, all variable definitions were based on ICD-10-CM codes which were derived from patients’ medical records. Disability status was determined based on eligibility for disability benefits, assessed through both age and health insurance coverage [15]. Hepatitis C and liver failure were included as both chronic comorbidities and complications.

Mental health diagnoses included bipolar disorder, depression, schizophrenia, and suicidal ideation. Substance use disorders included the following: alcohol, cannabis, cocaine, methamphetamines, opioids, and psychoactive substances. Alcohol use disorder (AUD) was defined as having one (or more) diagnosis codes associated with alcohol abuse or dependence, with or without intoxication (Further information on variable definitions can be found in the Supplementary Material).

Suicidal history was defined to include patients with documented thoughts of self-harm or a history of self-harm behavior. A “suicide attempt” was defined as an injury that was classified as either a suicide attempt or intentional self-harm, such as with a handgun or other means, and these were categorized as SBC cases [13].

Payer type was based on both the patient’s primary and, if applicable, secondary source of payment. There were four payer categories: Charity, Medicaid, Medicare, and Private. Patients were categorized by Hispanic ethnicity and then classified by race (Asian, Black, White, and Unknown). Health care outcomes included being treated in the intensive care unit (ICU), average length of stay, and mortality rate. In terms of geography, rural counties were defined as those with a population of less than 50,000 [16]. Mid-size cities were those counties with a population of between 50,000 and 200,000, while urban counties were those with a population of 200,000 or more.

Significant differences between OIS subjects and control subjects were identified using chi-square tests for categorical variables and *t*-tests for ordinal variables. Logistic regression models were used to identify predictors of an OIS. The statistical evaluation of variables, as well as a priori knowledge, was used to determine inclusion in the final model. Regression outcomes are reported as adjusted odds ratios (AORs), where appropriate. All confidence intervals were at the 95 percent level. All statistical analyses were performed using SPSS software version 28 (IBM, Armonk, NY, USA, 2021). No generative AI tools were used in the analysis, writing, or preparation of this manuscript. All analyses and interpretations were conducted solely by the author. This study was reviewed by Institutional Review Board (IRB) of the University of North Texas. The IRB determined that this study did not involve human subjects. All data were handled in compliance with applicable data privacy regulations.

## 3. Results

The youngest study subject was 13 and the oldest was 93. Persons aged 20–39 were at the greatest risk for an OIS. The risk of OIS was significantly lower for persons aged 12–19 and those 60 years and older. Among OIS subjects whose gender was known, only two (1.1%) were female. Blacks were at a greater risk for an OIS (*p* = 0.02). Non-Hispanic whites (*p* = 0.02) were found to be at a lower risk for an OIS, as shown in Table 1.

Nearly ten percent (9.6%) of OIS subjects belonged to a vulnerable population or had experienced recent trauma. Six OIS subjects (3.4%) were identified as persons experiencing homelessness (PEH). Five OIS subjects (2.8%) were disabled, including one who was wheelchair-dependent. OIS subjects were more likely to have recent family bereavement (1.1% vs. 0.1%; *p* < 0.001). Five subjects (2.8%) were diagnosed with post-traumatic stress disorder (PTSD).

OIS subjects were three times more likely to have a diagnosed mental illness compared to control subjects (24.3% vs. 8.4%; *p* < 0.001). Schizophrenia was the most common mental illness (11.3%), followed by bipolar disorder (9.6%). Major depression was not a statistically significant risk factor. Rates of suicidal history (5.6%) and suicide attempt/SBC (4%) were significantly higher among OIS subjects than control subjects (*p* < 0.001).

More than one-third (37.9%) of OIS subjects had one or more diagnoses related to drugs or alcohol, compared to 17.6% of control subjects (*p* < 0.001), as shown in Table 1. More than ten percent (11.3%) of OIS subjects had two or more SUDs. Methamphetamine use disorder (MUD) was the most common substance abuse issue, as it affected 14.7% of OIS subjects, compared to 2.5% of control subjects. The prevalence of AUD was ten percent or greater for both OIS subjects (14.1%) and control subjects (10.2%). Four study subjects (2.3%) had a blood alcohol concentration (BAC) of 0.1% or greater upon hospital admission. Opioid use disorder was more than twice as prevalent among OIS subjects as control subjects (4.5% vs. 1.7%; *p* < 0.001). Cocaine UD was also significantly higher among OIS subjects (7.3% vs. 2.2%; *p* < 0.001). In terms of chronic disease and clinical outcomes, OIS subjects were more than twice as likely to have hepatitis C (*p* < 0.01) and liver failure (*p* < 0.001) compared to control subjects. Mortality rates were more than three times higher for OIS subjects compared to control subjects (11.3% vs. 3.2%; *p* < 0.001). The average length of stay due to trauma was almost twice as long for OIS subjects (12.3 days vs. 6.9 days; *p* < 0.001). Fifteen percent of OIS subjects had a length of stay of 20 days or longer. OIS subjects were more than four times more likely to be physically restrained during their hospital stay (7.3% vs. 1.5%; *p* < 0.001).

To summarize, more than half (53.7%) of OIS subjects had at least one risk factor compared to 26.9% of control subjects (*p* < 0.001). Risk factors included mental illness, substance use, suicidal ideation, recent trauma, and a member of a vulnerable population.

As shown in Table 2, the most significant predictors of OIS were suicide attempt/SBC (AOR = 46.1; CI: 18.2, 116.3), recent bereavement (AOR = 8.45; CI: 1.8; 39.6), schizophrenia (AOR = 2.72; CI: 1.62, 4.58), and Methamphetamine UD (AOR = 3.48; CI: 2.22, 5.45). The risk of an OIS incident was about twice as high for persons aged 20 to 39 (AOR = 2.08; 95% CI: 1.49–2.90). Elderly persons, aged 60 and older, were at a reduced risk for an OIS incident (AOR = 0.24; CI: 0.11–−0.50). Having health insurance was associated with a lower risk for OIS. This included persons with commercial insurance (AOR = 0.27; CI: 0.17–−0.45) and those covered by Medicaid (0.61; CI: 0.11–−0.93). Race and ethnicity were not found to be significant predictors of OIS incidents.

## 4. Discussion

The most significant predictor of OIS was “suicide attempt,” commonly referred to as “suicide by cop” (SBC). There is extensive literature on the psychological underpinnings and characteristics of SBC, as a distinct category of OIS [17]. For the law enforcement officers involved in SBC, this can be an especially stressful and demoralizing form of shooting trauma [18]. In practice, the risk factors for SBC and OIS often overlap, including recent trauma, mental illness, and substance use. In either case, there is rarely a single, definitive cause. Rather there is typically a confluence of factors. In what follows, we will examine each of these risk factors in turn before revisiting the topic of SBC.

In terms of demographics, males in their twenties and thirties were at the greatest risk for an OIS, and this is consistent with the previous literature [19]. Age was also found to be a protective factor, such as for persons younger than 20 and those age 60 and older. Many previous studies in this area have included information on demographic variables, such as age, sex, race, and ethnicity. However, due to a lack of data availability, very few studies have included information on vulnerable population status, such as homelessness, disability, and recent trauma. Six OIS subjects (3.4%) were persons experiencing homelessness, and four of these had mental illness. PEH are likely to have more frequent contact with law enforcement due to factors such as mental illness, substance use, and reliance on public spaces [20]. A previous study found that PEH comprised 1.7 percent of 812 fatal OIS incidents [3]. To our knowledge, this was the only previous study that examined the impact of OIS on the homeless population.

This study found that bereaved persons were seven times more likely to experience an OIS incident. These subjects also had mental illness (major depression, schizophrenia). Previous descriptive studies have found that, in many cases of OIS, there was a personal crisis (such as divorce, job loss, serious medical diagnosis, or foreclosure on a house) within the two weeks preceding the incident [17,18,21]. To our knowledge, this is the first study to quantify the effect of a recent trauma, such as bereavement, on the risk of an OIS. In addition, 2.8% of OIS subjects had PTSD.

Persons with schizophrenia were found to be 2.7 times more likely to experience an OIS, compared to control subjects. This is consistent with prior research. One study found that persons diagnosed with schizophrenia on Medicaid were more than three times as likely to die from an OIS incident. They were also five times more likely to die from suicide [22]. Another study found that persons with schizophrenia were more likely to sustain serious injuries due to legal interventions [23]. These findings align with the literature emphasizing the vulnerability of individuals with serious mental illness during encounters with law enforcement [24]. Factors contributing to this elevated risk may include impaired communication, confusion, difficulty responding to commands, heightened paranoia, or behavior perceived as threatening. In many cases, these individuals are in crisis or experiencing psychosis, which can escalate routine police encounters into violent confrontations. Among persons with schizophrenia (*n* = 735), the rate of homelessness was 24%, compared to 1.8% among those without schizophrenia. Bipolar disorder and major depression were not directly linked to OIS incidents. However, both of these were associated with suicidal behavior and substance use.

Methamphetamines were found to be the most dangerous substance in terms of OIS incidents. Persons with methamphetamine use disorder (MUD) were 2.5 times more likely to experience an OIS. MUD was more prevalent among white subjects (23%) compared to non-white subjects (10%; *p* < 0.05). Persons with MUD were five times as likely to have hepatitis C (11.5% vs. 2%; *p* < 0.05), and hepatitis C was also associated with liver failure (*p* < 0.05) and has also been linked to injection drug use [25]. The prevalence of hepatitis C was also significantly higher among persons with schizophrenia (*p* < 0.01).

Within the control group (*n* = 33,529), MUD increased over time, rising from 1% in 2016 to 3% in 2023 (*p* < 0.001). This troubling trend mirrors the national rise in methamphetamine use [26]. In a study of 350 persons with MUD, more than half (56%) reported that methamphetamine use led to violent behavior [27]. MUD also has profound medical consequences and often results in long-term cognitive and neurological deficits [26]. In addition, there has been a significant increase in the availability and potency of the supply of illicit methamphetamines in recent years, further exacerbating these issues [25].

This study also found a significant co-occurrence of MUD and schizophrenia, a relationship well documented in the literature [28]. More than one-third (35%) of study subjects with schizophrenia (*n* = 20) also had MUD. Methamphetamine use is likely to exacerbate schizophrenia symptoms such as paranoia and psychosis, potentially leading to aggressive or erratic behavior. Prolonged methamphetamine use has also been associated with persistent psychosis that resembles schizophrenia [28]. Moreover, the risk of an officer-involved shooting (OIS) was found to be 2.67 times greater for persons with both schizophrenia and MUD (AOR = 2.67; CI: 1.04, 6.83; *p* < 0.05), as compared to those with schizophrenia who do not have MUD. These findings highlight the compounded risks associated with the co-occurrence of MUD and schizophrenia.

Even though 14 percent of OIS subjects had alcohol use disorder, AUD was not a significant predictor of OIS incidents. Within the control group, AUD exceeded ten percent, as alcohol consumption is a contributing factor in many car accidents. One previous study of 110 OIS incidents found that 26 percent of subjects were under the influence of alcohol, based on toxicology reports [13]. Another study found AUD to be a risk factor for serious injury due to legal intervention [23].

All of the risk factors discussed so far also apply to SBC cases. A previous study found that SBC cases constitute 18 percent of all fatal OIS incidents [3]. In the present study, only four percent (*n* = 7) of all OIS incidents were classified as SBC. The true prevalence of SBC cases is likely to be significantly higher because most SBC cases result in mortality prior to hospital admission. Six of the seven SBC cases had mental illness, including bipolar disorder (*n* = 4) and major depression (*n* = 2). Five of the subjects had one or more self-inflicted gunshot wounds. Three persons had a documented history of one or more suicide attempts. SBC cases also differed from non-SBC cases in several important ways. These subjects were older (45 vs. 36; *p* < 0.05), more likely to be white (71% vs. 33%; *p* < 0.05), and more likely to reside in a medium-size city (43% vs. 16%; *p* < 0.05). There was one fatality among the SBC cases and three subjects were discharged to a psychiatric hospital.

This study used data from one state. Texas is the second largest state in the US, with a population of about 30 million. While large and diverse, Texas has unique demographics and law enforcement practices that may not fully represent those of other states. Therefore, caution is advised when generalizing these results to other states or populations. Two studies used national data to rank all 50 states in terms of the frequency of OIS incidents and the completeness of OIS reporting [29,30]. Texas is also one of only three states that mandates the disclosure of OIS incidents, a practice that enhances transparency and ensures that law enforcement data are publicly available [7].

There are several limitations in using hospital discharge data to study OIS. First, the majority of persons who die due to OIS never make it to the hospital. Therefore, these cases are not included in the database. Likewise, persons who are treated in the emergency department and released are not included. There is no information on the reason for the OIS, such as whether the suspect had a weapon or posed an immediate threat. According to previous research, the majority of OIS incidents involve an armed suspect. One study of 812 fatal OISs found that 82% of suspects were reportedly armed (or assumed armed) with a deadly weapon [3]. One previous study found that persons with mental illness were more likely to be armed with a knife than with a firearm, and that they were more likely to be shot in their own homes [2]. In addition, some of the OISs may have been misclassified as homicides or justifiable homicides [31].

Despite these challenges, hospital discharge data remain a valuable resource for analyzing both fatal and nonfatal OIS incidents. Hospital data contain detailed information on the patient’s medical history, including chronic diseases, vulnerable population status, mental health diagnoses, and substance use disorders. The data also contain information on OIS incidents by other than law enforcement (e.g., security guards) that are legally authorized to use force. There is evidence that the validity and completeness of hospital data have improved in recent years due to various factors. These include the transition to ICD-10-CM, the increased use of Electronic Health Records (EHRs), and the passage of reporting requirements at the state level [32]. In addition, some hospital inpatient databases (though not all) contain “Z codes” for public health surveillance and the social determinants of health (Z55–Z65) [33]. Some examples of Z codes include homelessness (Z59.0), personal history of self-harm (Z91.5), and dependence on wheelchair (Z99.3). A recent study examined the use of the code for homelessness (“Z59.0”) in hospital records [34]. Although the code had a high positive predictive value, its sensitivity was low. Thus, the true prevalence of homelessness among OIS subjects was likely higher than what was reported in the database.

This study provides evidence that health insurance, particularly commercial insurance and Medicaid, may be protective against OIS incidents. Commercial insurance could be a proxy for stable employment, of either the patient or a family member, such as a spouse or parent. Medicaid enrollment was found to be significantly higher for persons with schizophrenia (33%) and bipolar disorder (30%) compared to those without mental illness (17%). This highlights Medicaid’s role in providing access to critical services, such as psychotropic medications and case management. Reductions in Medicaid funding or eligibility may impede access to these services. Over time, untreated mental illness will lead to more acute psychiatric events and subsequent police encounters. Because Texas is a non-expansion state, this also may limit Medicaid access for low-income adults. While not captured in the database, marital status also plays a significant role in both social stability and Medicaid eligibility. Further research is needed to better understand these relationships.

Law enforcement agencies have increasingly recognized the need to implement changes to reduce deaths among persons with mental illness [2]. Crisis intervention team (CIT) training has been widely adopted as a means of preparing officers to handle such situations [35]. By improving officers’ understanding of mental illness, CIT training helps them identify behavior as symptoms rather than signs of violent resistance. The program emphasizes de-escalation techniques and connecting persons with appropriate support services as an alternative to jail. There are more than 1000 active CIT programs operating in forty-nine states and four countries [36].

While CIT training is widely implemented, its actual effectiveness for reducing adverse outcomes has been the subject of ongoing debate [24]. A recent systematic review on CIT program effectiveness found some evidence of benefits [36]. CIT training was shown to improve officer-level outcomes, such as increased job satisfaction and a perceived reduction in the use of force. For CIT programs to be effective, it is essential to prioritize those psychiatric conditions and substances that pose the greatest risk for OIS incidents, based on the most current evidence.

## 5. Conclusions

This study advances the literature on officer-involved shootings (OISs) by disaggregating mental illness into specific psychiatric diagnoses—such as bipolar disorder and schizophrenia—rather than treating it as a single category. It also distinguishes between individual substances, including alcohol, cocaine, methamphetamines, and opioids, instead of grouping them under general substance use. This level of diagnostic specificity enables a more nuanced understanding of individual risk factors and their potential interactions, such as the co-occurrence of schizophrenia and methamphetamine use. Moreover, this study found a high degree of inter-correlation between the main variables of interest and covariates associated with marginalized groups, including disability, hepatitis C, housing instability, PTSD, and recent trauma. This approach helps identify individuals at the highest risk for an OIS and supports the development of targeted prevention and intervention strategies. These findings underscore the preventable nature of many OIS incidents, especially those that involve untreated mental illness, substance use disorders, and recent trauma. Addressing the root causes of OIS incidents will likely require interdisciplinary collaboration among law enforcement, public health agencies, and social services.

## Figures and Tables

**Table 1:** Patient Characteristics of Subjects of Officer-Involved Shooting. Compared to Control Group in Car Accidents.

		Officer Inv.	Controls		
		Shooting	Car Accidents		
Patients		(*n* = 177)	(*n* = 33,539)	χ^2^	*p*-Value
Age					
	Age 12–19	4.5%	8.3%		
	Age 20–39	66.7%	36.1%		
	Age 40–59	23.2%	25.8%		
	Age 60+	4.5%	26.5%		
	Age Missing	1.1%	3.2%	83.4	0.000
Race/Ethnicity					
	Asian	0.6%	2.1%		
	Black, non-Hispanic	21.5%	15.3%		
	Hispanic	30.5%	31.7%		
	White, non-Hispanic	34.5%	43.4%		
	Race Missing	13.0%	7.7%	16.2	0.000
Vulernable Pop. & Recent Trauma					
	Disabled	2.8%	4.1%	0.44	>0.10
	Wheelchair Dependent	0.6%	0.1%	0.55	>0.10
	Homeless, Person Exp.	3.4%	2.4%	0.37	>0.10
	Post-Traumatic Stress (PTSD)	2.8%	1.2%	3.56	0.06
	Recent Bereavement	1.1%	0.1%	9.15	<0.001
	One or More	9.6%	7.5%	0.84	>0.10
Mental Illness					
	Bipolar Disorder	9.6%	2.6%	31.2	<0.001
	Depression	5.1%	4.5%	0.03	>0.10
	Schizophrenia	11.3%	2.1%	65.3	<0.001
	One or More	24.3%	8.4%	55.2	<0.001
Suicide/Self-Harm					
	Suicidal History	5.6%	0.9%	36.6	<0.001
	Suicide Attempt (Current)	4.0%	0.1%	276.3	<0.001
	Total	7.9%	1.0%	76.9	<0.001
Substance Use Disorders					
	Alcohol Use Disorder	14.1%	10.2%	2.99	0.084
	Cannabis UD	11.3%	4.4%	19.7	<0.001
	Cocaine UD	7.3%	2.2%	19.9	<0.001
	Methamphetamine UD	14.7%	2.5%	100.5	<0.001
	Opiod UD	4.5%	1.7%	7.17	0.003
	Psychoactive, other	3.4%	1.0%	7.98	0.001
	One or More	37.9%	17.3%	49.7	<0.001
Chronic Comorbidities					
	Hepatitis C	3.4%	1.2%	7.17	0.007
	Liver Failure	2.8%	0.5%	7.98	0.005
Clinical Outcomes					
	Mortality	11.3%	3.2%	34.17	<0.001
	Length of Stay (Days)	12.3	6.9		<0.001
	Intensive Care Unit	71.8%	49.6%	33.47	<0.001
Health Insurance					
	Charity	63.3%	39.7%	41.95	<0.001
	Medicaid	15.3%	17.1%	0.30	>0.10
	Medicare	4.5%	13.3%	11.23	<0.001
	Commercial (Private)	10.2%	30.9%	35.07	<0.001

**Table 2:** Predictors of officer-involved shooting based on subject characteristics.

		Odds	Lower	Upper
Subjects (N = 177)		Ratio	Limit	Limit
Age				
	Age 20–39	2.08	1.49	2.90
	Age 60+	0.24	0.11	0.50
Health Ins.				
	Medicaid	0.61	0.11	0.93
	Commercial	0.27	0.17	0.45
Mental Illness				
	Schizophrenia	2.72	1.62	4.58
Recent Trauma				
	Bereavement	8.45	1.81	39.57
Substance Use				
	Methamphetamine UD	3.48	2.22	5.45
Suicide/Self-Harm				
	Suicide Attempt/SBC	46.1	18.2	116.3

*p*-value for Medicaid = 0.02; all other *p*-values < 0.01.

## Data Availability

The Texas Inpatient Public Use Data File used in this study was licensed by the authors, and the data use agreement signed by the authors disallows them from sharing the data themselves. However, these data are publicly available from the Center for Health Statistics at the Texas Department of State Health Services. The request form (which includes the data use agreement) is available from the DSHS website (https://www.dshs.texas.gov, accessed on 12 June 2025).

## Supplementary Materials

The following supporting information can be downloaded at: https://www.mdpi.com/article/10.3390/ijerph22060945/s1. References [37,38] are cited in the Supplementary Materials.

## Abbreviations

The following abbreviations are used in this manuscript:

AUD	Alcohol use disorder
CIT	Crisis intervention team
MUD	Methamphetamine use disorder
OIS	Officer-involved shooting
PEH	People experiencing homelessness
