# Supplementary material for: The Role of Mental Health, Recent Trauma, and Suicidal Behavior in Officer-Involved Shootings: A Public Health Perspective
Source: Int J Environ Res Public Health. 2025 Jun 17;22(6):945. doi: 10.3390/ijerph22060945 (PMC12192678; doi:10.3390/ijerph22060945)
Supplement: Supplementary file 1 [file ijerph-22-00945-s001.zip › ijerph-3653225-supplementary.pdf]

Supplementary Material

The following ICD10-CM Codes were used in this analysis.

Alcohol

F1010	F10120	F10121	F10129	F10130	F10131	F10132	F10139	F1014	F10150
F10151	F10159	F10180	F10181	F10182	F10188	F1019	F1020	F10220	F10221
F10229	F10230	F10231	F10232	F10239	F1024	F10250	F10251	F10259	F1026
F1027	F10280	F10281	F10282	F10288	F1029	F10920	F10921	F10929	F10930
F10931	F10932	F10939	F1094	F10950	F10951	F10959	F1096	F1097	F10980
F10981	F10982	F10988	F1099	G312	G621	I426	K2920	K2921	K700
K7010	K7011	K702	K7030	K7031	K7040	K7041	K709		

Bipolar Disorder

F0633	F0634	F3010	F3011	F3012	F3013	F302	F303	F308	F309
F310	F3110	F3111	F3112	F3113	F312	F3130	F3131	F3132	F314
F315	F3160	F3161	F3162	F3163	F3164	F3171	F3173	F3175	F3177
F3181	F3189	F319	F340						

Cannabis

F1210	F12120	F12121	F12122	F12129	F1213	F12150	F12151	F12159	F12180
F12188	F1219	F1220	F12220	F12221	F12222	F12229	F1223	F12250	F12251
F12259	F12280	F12288	F1229	F1290	F12920	F12921	F12922	F12929	F1293
F12950	F12951	F12959	F12980	F12988	F1299	T407X1A	T407X2A	T407X3A	T407X4A
T407X5A									

Car Accidents

V405XXA	V407XXA	V425XXA	V4302XA	V4312XA	V4352XA	V4352XD	V4353XA	V4353XD	V4354XA
V4362XA	V4362XD	V4363XA	V4372XA	V445XXA	V446XXA	V449XXA	V451XXA	V466XXA	V470XXA
V471XXA	V475XXA	V476XXA	V479XXA	V480XXA	V481XXA	V482XXA	V482XXD	V484XXA	V485XXA
V485XXD	V486XXA	V493XXA	V493XXD	V4940XA	V4940XD	V4940XS	V4949XA	V4950XA	V4959XA
V4960XS	V4988XA	V499XXA	V499XXD	V499XXS	V500XXA	V535XXA	V536XXA	V545XXA	V546XXA
V570XXA	V575XXA	V576XXA	V580XXA	V581XXA	V582XXA	V584XXA	V585XXA	V586XXA	V5940XA
V5950XA	V5988XA	V599XXA							

Cocaine

F1410	F1411	F14120	F14121	F14122	F14129	F1413	F1414	F14150	F14151
F14159	F14188	F1419	F1420	F1421	F14220	F14221	F14222	F14229	F1423
F1424	F14250	F14251	F14259	F14288	F1429	F1490	F14920	F14921	F14922
F14929	F1493	F1494	F14950	F14951	F14959	F1499			

Depression

F0631	F0632	F0634	F320	F321	F322	F323	F324	F328	F3281
F3289	F329	F330	F331	F332	F333	F3341	F338	F339	F341

Hepatitis C

B1710	B1711	B182	B1920	B1921

Homeless

Z590	Z5901	Z5902

Liver Failure

K7200	K7201	K7210	K7211	K7290	K7291

Methamphetamines and Other Stimulants

F1510	F1511	F15120	F15121	F15122	F15129	F1513	F1514	F15150	F15151
F15159	F15180	F15181	F15182	F15188	F1519	F1520	F1521	F15220	F15221
F15222	F15229	F1523	F1524	F15250	F15251	F15259	F15280	F15281	F15282
F15288	F1529	F1590	F15920	F15921	F15922	F15929	F1593	F1594	F15950
F15951	F15959	F15980	F15981	F15982	F15988	F1599	T43621A		

Opioids

F1110	F11120	F11121	F11122	F11129	F1113	F1114	F11150	F11151	F11159
F11181	F11182	F11188	F1119	F1120	F11220	F11221	F11222	F11229	F1123
F1124	F11250	F11251	F11259	F11281	F11282	F11288	F1129	F1190	F11920
F11921	F11922	F11929	F1193	F1194	F11950	F11951	F11959	F11981	F11982
F11988	F1199	T400X1A	T400X1D	T400X2A	T400X2D	T400X3A	T400X3D	T400X4A	T400X4D
T400X5A	T400X5D	T401X1A	T401X1D	T401X2A	T401X2D	T401X3A	T401X3D	T401X4A	T401X4D
T402X1A	T402X1D	T402X2A	T402X2D	T402X3A	T402X3D	T402X4A	T402X4D	T402X5A	T402X5D
T403X1A	T403X1D	T403X2A	T403X2D	T403X3A	T403X3D	T403X4A	T403X4D	T403X5A	T403X5D
T40411A	T40411D	T40412A	T40412D	T40413A	T40413D	T40414A	T40414D	T40415A	T40415D
T40421D	T40422A	T40422D	T40423A	T40423D	T40424A	T40424D	T40425A	T40425D	T40491A
T40491D	T40492A	T40492D	T40493A	T40493D	T40494A	T40494D	T40495A	T40495D	T404X1A
T404X1D	T404X2A	T404X2D	T404X3A	T404X3D	T404X4A	T404X4D	T404X5A	T404X5D	T40601A
T40601D	T40602A	T40602D	T40603A	T40603D	T40604A	T40604D	T40605A	T40605D	T40691A
T40691D	T40692A	T40692D	T40693A	T40693D	T40694A	T40694D	T40695A	T40695D	

Post Traumatic Stress Disorder

F4310	F4311	F4312

Psychoactive Drugs

F1910	F1911	F19120	F19121	F19122	F19129	F19130	F19131	F19132	F19139
F1914	F19150	F19151	F19159	F1916	F1917	F1918	F1919		

Schizophrenia

F200	F201	F202	F203	F205	F2081	F2089	F209	F21	F22
F23	F24	F250	F251	F258	F259	F28	F29		

Suicidal Behavior and Intentional Self-Harm

R45851	Z915	Z9151	Z9152	X71–X83	T1491XA

Trauma: Disappearance or Death of Family Member

Z634

Legal Intervention

Y35003A; Y35023A; Y35093A.

Variable Definition

There are no specific codes in ICD-10-CM for methamphetamine abuse, dependence, or poisoning. Hence we used codes classified under 'other stimulant use.' Prior research has found that these codes have a high positive predictive value (> 85%) for identifying methamphetamine use [37, 38]. However, they may also capture the use of other stimulants, such as prescription amphetamines.
